# The roles and patterns of critical care pharmacists: a literature review and practical operation model in China

**DOI:** 10.3389/fphar.2024.1439145

**Published:** 2024-11-06

**Authors:** Chunyan Wei, Jinhan He, Jingyi Zhang, Huifang Shan, Aidou Jiang, Ying Liu, Guanghui Chen, Chaoran Xu, Linchao Wang, Xiaofen Shao, Wanhong Yin

**Affiliations:** ^1^ Department of Pharmacy, West China Hospital, Sichuan University, Chengdu, China; ^2^ West China School of Pharmacy, Sichuan University, Chengdu, Sichuan, China; ^3^ Department of Pharmacy, Xiangtan Central Hospital, Xiangtan, China; ^4^ Department of Pharmacy, The Third People’s Hospital of Chengdu, Chengdu, China; ^5^ Department of Pharmacy, The First People’s Hospital of Jining, Jining, China; ^6^ Department of Pharmacy, Ziyang Central Hospital, Ziyang, China; ^7^ Department of Critical Care Medicine, West China Hospital, Sichuan University, Chengdu, China; ^8^ West China School of Clinical Medical College, Sichuan University, Chengdu, China

**Keywords:** critical care pharmacists, medication management, critically ill patients, optimize pattern, medical rounds

## Abstract

Drug-related problems (DRPs) are prevalent in critically ill patients and may significantly increase mortality risks. The participation of critical care pharmacists (CCPs) in the medical team has demonstrated a benefit to healthcare quality. Research indicates that CCP medication order evaluations can reduce DRPs, while their participation in rounds can reduce adverse drug events and shorten hospital stays. Pharmacist medication reconciliation often proves more effective than physicians, and CCPs play a crucial role in antimicrobial management and reducing treatment costs. Despite these benefits, there is a noticeable lack of practical guidance for implementing CCP roles effectively. Their workflow heavily influences the efficiency of CCPs. Integrating results from the literature with our practical experience, we have detailed workflows and critical entry points that CCPs can refer to. Pharmacists should be proactive rather than passive consultants. Pre-round medication order evaluations are crucial for determining the depth of a pharmacist’s involvement in patient care. These evaluations should cover the following aspects: medication indication, dosage, treatment duration, detection of DRPs, implementation of therapeutic drug monitoring, dosing of sedatives and analgesics, and pharmaceutical cost containment. Beyond identifying medication issues, a primary task during rounds is gathering additional information and building trust with the medical team. Post-round responsibilities for CCPs include patient and caregiver education on medication, medication reconciliation for transitioning patients, and follow-up care for post-ICU patients. Establishing a rationalized and standardized workflow is essential to minimize daily work omissions and maximize the pharmacist’s value. A multidisciplinary pharmacist-led team can significantly promote the rational use of antibiotics. Participation in post-ICU outpatient follow-ups can reduce drug-induced injuries after discharge. This review provides a detailed overview of the tasks performed by CCPs before, during, and after medical rounds, serving as a valuable reference for establishing an efficient workflow for CCPs.

## 1 Introduction

Drug-related problems (DRPs) are more prevalent in intensive care units (ICUs) than in general care units (Cullen et al., 1997; Singh et al., 2011) due to a combination of factors. Critically ill patients receive twice as many medications as non-critically ill patients, resulting in a higher probability of adverse drug events (ADEs) (Cullen et al., 1997). ICU patients are more prone to experiencing drug-drug interactions (DDIs), and the occurrence of multiple organ function impairment in ICU patients is mutually causative with inappropriate drug therapy (Bakker et al., 2021; Moyen et al., 2008). Previous studies have found that the incidence of medication errors (MEs) in adult ICU patients ranges from 1.2 to 947 errors per 1,000 patient ICU days, with a median of 106 errors per 1,000 ICU days (Kane-Gill and Weber, 2006). MEs are a significant cause of morbidity and mortality in patients. Approximately 19% of MEs in the ICU are life-threatening, and nearly 42% are clinically significant enough to require additional life-sustaining treatment. Overall, critically ill patients are at higher risk of harm from DRPs and ADEs due to frequent and more severe medication-related events. Therefore, medication safety and efficacy are crucial for patients with critical illness.

The intervention of critical care pharmacists (CCPs) is valuable in preventing the occurrence of DRPs in the ICU (Heselmans et al., 2015; Malfará et al., 2018; Stollings et al., 2018; Stollings et al., 2023). Previous systematic reviews and meta-analyses have shown that CCP intervention may significantly reduce preventable ADEs and prescribing errors (Wang et al., 2015). Pharmacist intervention as part of the multidisciplinary ICU team was significantly associated with reduced mortality and length of stay in the ICU (Lee et al., 2019). Although CCPs are not revenue generators, they help avoid ICU-related costs. Economic analyses have consistently indicated a high return on investment, with the predicted cost-avoidance to CCP salary ranging from $3.3:1 to $9.6:1 (MacLaren et al., 2008; Sikora, 2023). Furthermore, ICUs without CCPs were found to have significantly higher mortality rates and longer lengths of ICU stay, with the economic return on investment for a CCP estimated at 25:1 (MacLaren et al., 2008; Sikora, 2023). Increasing attention is being paid to CCPs. An article published in Intensive Care Medicine (ICM) in January 2024 described ten reasons an ICU requires pharmacists (McKenzie et al., 2024). This article emphasizes the value of clinical pharmacists, including improving patient outcomes, ensuring patient safety, optimizing patient treatment, and improving cost-benefit.

Given the complex and specialized environment of the ICU, the significance of CCPs does not require excessive emphasis. However, the current academic literature does not precisely depict the operational modalities CCPs use. This element is deemed pivotal in our view. Relevant work patterns enable CCPs to identify areas where value can be applied and optimize their utilization. The article published in ICM also emphasizes that the initial step in establishing and expanding the ICU pharmacist profile should involve the development of a comprehensive white paper describing the roles and key priorities of ICU pharmacy professionals (McKenzie et al., 2024). The “Position Paper on Critical Care Pharmacy Services: 2020 Update” was published in Critical Care Medicine (Lat et al., 2020), providing an updated version of the previously published article in 2000 (Society of Critical Care Medicine and the American College of Clinical Pharmacy et al., 2000). The article lists 82 recommendations related to critical care pharmacist duties and pharmacy services, quality improvement, and research and scholarship domains. The implementation of each of these tasks still raises some questions. No literature can tell us how CCPs should be structured to serve as many patients as possible. Therefore, this article aims to summarize the models and patterns in the relevant literature on CCPs and to find valuable entry points for CCPs. At the same time, the CCP’s working pattern in our hospital is introduced to provide a reference for other institutions.

## 2 Methods

We conducted a comprehensive search in the Pubmed database to retrieve relevant scientific literature pertaining to the assessment the work patterns and subsequent impact of CCPs. The Search terms include “ICU”, “intensive care unit”, “critical care”, “pharmacist”, “clinical pharmacist”, “critical care pharmacist”. The contribution of CCPs in medication orders evaluation, ward rounds, medication reconciliation, antibiotic management and cost control were reviewed. Then, we reviewed the patterns of CCPs in our hospital. We present a comprehensive range of essential tasks that CCPs can undertake before, during, and after rounds. Additionally, we highlight specialized projects in which CCPs can actively engage, with the aim of optimizing the value contributed by pharmacists.

## 3 Results

### 3.1 Review of the literature on the roles and patterns of CCPs

**FIGURE 1:**
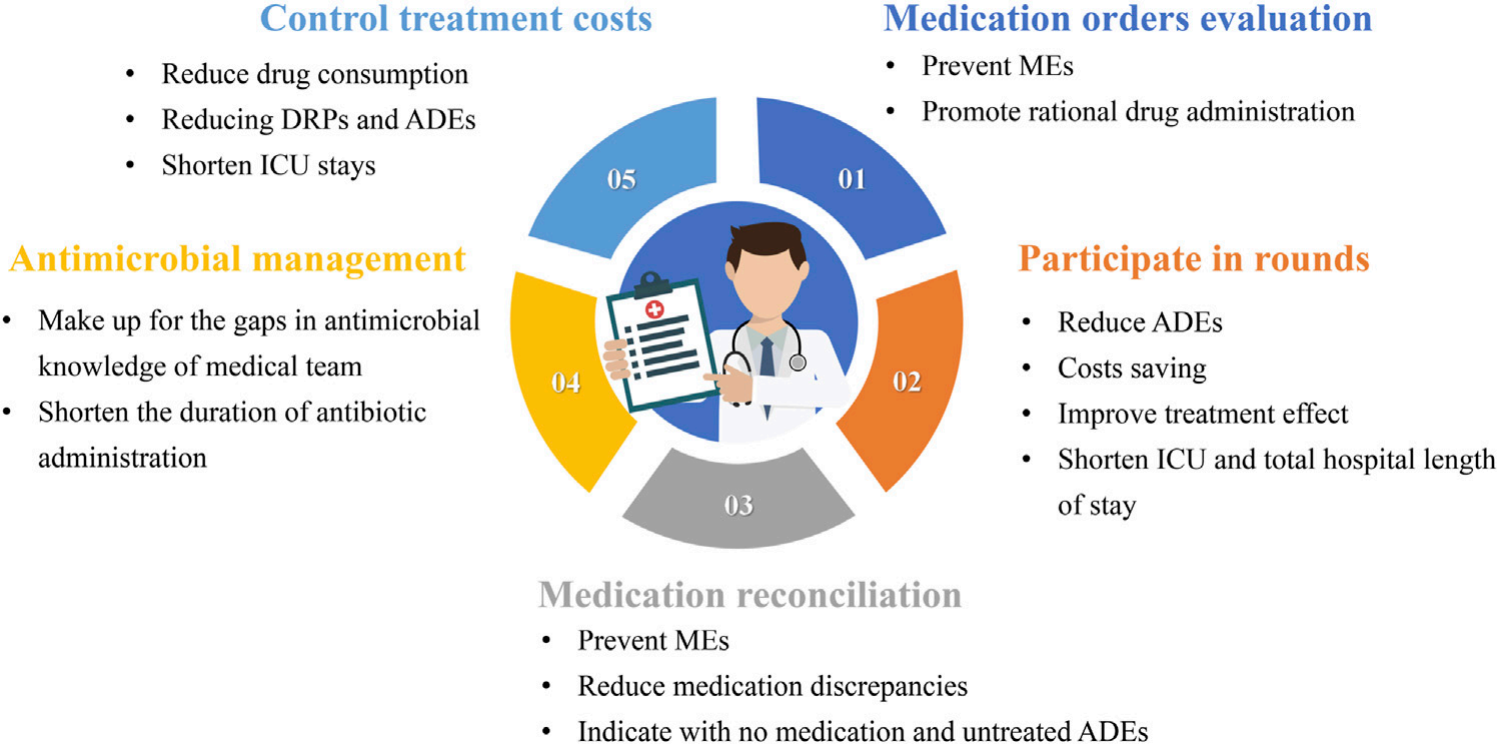
The value of the various types of work of critical care pharmacists MEs: medication errors; ADEs: adverse drug events; ICU: Intensive care unit; DRPs: drug-related problems.

The work of CCPs should be patient centered. A previous review introduced three practice patterns of CCPs (Brilli et al., 2001). In the first model, CCPs retrospectively evaluate medication orders but do not attend ICU rounds. In the second model, CCPs are assigned to a satellite pharmacy in the ICU, with simultaneous responsibilities including dispensing medications, prospectively evaluating medication orders, and attending ICU rounds. In the third model, CCPs specialize in direct patient care responsibilities, including attending daily rounds, obtaining medication histories, and prospectively evaluating drug therapy. Consultative services of CCPs in pharmacotherapy, nutrition support, or pharmacokinetics/pharmacodynamics (PK/PD) may be available as an added service to any of the practice patterns. The involvement of pharmacists in these three models is different. Although no research proves which model benefits patients more, the third model, which is more patient-centered in delivering pharmaceutical care, may be more beneficial. As shown in Figure 1, each type of pharmacist’s work has its specific value.

#### 3.1.1 Medication orders evaluation (MOE)

Reducing MEs is the most fundamental goal of CCPs, and this can be achieved in several ways. Evaluating medication orders is the most basic task and entails several aspects. The first aspect is determining whether the medication is suitable, and the second is identifying any MEs. The practice guidelines for safe drug use in the ICU state that when pharmacists do not verify prescriptions before administration, it can lead to wrong medication administration, incorrect doses, wrong times, and wrong formulations (Kane-Gill et al., 2017). Federal regulations require pharmacists to conduct medication administration observations, record nurse activities, and coach nurses on safe medication administration techniques. Most studies on the effectiveness of medication order evaluations have focused on non-ICU patients. The findings suggest clinical pharmacists can reduce MEs in pediatric patients using antibiotics by evaluating patients’ medications based on DRPs, such as drug interactions, side effects, and prescribing errors (Özdemir et al., 2021). We believe that this result can be extrapolated to ICU patients.

#### 3.1.2 Participation in rounds

Are rounds necessary for CCPs? An article published in JAMA in 1999 demonstrated that pharmacist participation in physician rounds significantly reduced ADEs (Leape et al., 1999). The results showed that when pharmacists conducted rounds with the ICU team, the rate of preventable ordering ADEs decreased from 10.4 per 1,000 patient days to 3.5 per 1,000 patient days. Similarly, pharmacist participation in rounds of ICU patients receiving continuous venovenous hemofiltration (CVVH) resulted in cost savings and a 2.36-fold reduction in antimicrobial-related ADEs (Jiang et al., 2014). Another study showed that pharmacists participating in multidisciplinary management teams for pain, agitation, and delirium in the ICU led to fewer hours of patient exposure to continuous sedation, reductions in ICU and total hospital stay, and reduced hospital and drug costs (Louzon et al., 2017).

Rounds are a crucial medical practice that fosters communication between the pharmacist, the medical team, and the patient. Pharmacists must participate in rounds to fully obtain comprehensive treatment information, understand treatment goals, and make the most reliable judgments regarding the rationality of drug treatment. The participation of CCPs in rounds is a systematic work that involves answering questions from doctors or nurses and rationalizing medication evaluation, including drug selection, dose adjustment, and adverse reaction identification. It is important to determine the specific procedures for rounds, and we will detail the procedures for reference in the following sections.

#### 3.1.3 Medication reconciliation

It is well known that transitions of care are generally risky processes as they may generate MEs. Pharmacists’ medication reconciliation (MR) has been shown to prevent MEs (Bosma et al., 2018; Martínez Pradeda et al., 2023). An observational study showed that 34.29% of patients discharged from the ICU to a hospital ward had at least one discrepancy, and 18.14% had at least one reconciliation error (Martínez Pradeda et al., 2023). Compared to physician-led reconciliation, pharmacist-led reconciliation resulted in fewer medication discrepancies, particularly regarding indications without medication and untreated ADEs (El Hadidi et al., 2022).

A referential working model for MR is that CCPs reconcile the patient’s medication history within 24–48 h after ICU admission, resulting in an optimal medication history that is presented to the ICU physician. At ICU discharge, the CCPs reconcile the prescribed ICU medication history with the ICU physician, forming an ICU discharge medication list with medication prescription recommendations for the general ward physician (Bosma et al., 2017).

#### 3.1.4 Antimicrobial management

Antimicrobial therapy is a double-edged sword. On the one hand, proper and reasonable use can save the lives of patients with sepsis; on the other hand, overuse may bring the risk of bacterial resistance and adverse drug reactions (Kollef et al., 2021). Due to the large number of sepsis and septic shock patients in the ICU, the management of antibiotics in the ICU has been a topic of concern (Murphy et al., 2022). Research shows that the knowledge of antibiotic PK/PD among ICU physicians is generally insufficient (Mao et al., 2022). Therefore, participation in antimicrobial management can be a valuable entry point for CCPs.

The results of a systematic review showed that pharmacists’ participation in a multidisciplinary team in the treatment of patients with sepsis and septic shock could shorten the duration of antibiotic administration without increasing patient mortality (Atkins et al., 2023). Pharmacists’ participation in antimicrobial management should be diversified. This can include assessing antimicrobial guidelines during rounds, training doctors and nurses on antimicrobial knowledge, providing antimicrobial consultation, and participating in multidisciplinary teams related to antimicrobial drugs.

#### 3.1.5 Control treatment costs

ICU treatment is costly due to the use of multiple drugs, mechanical ventilation, and extracorporeal life support (Lefrant et al., 2015). Despite the small proportion of ICU beds relative to all hospital services, the ICU represents a significant cost to the hospital (Bruyneel et al., 2023). CCP efforts can reduce costs by decreasing drug consumption, reducing DRPs and ADEs, and shortening ICU stays (Aljbouri et al., 2013; Polat et al., 2022; Rech et al., 2021; Zaidi et al., 2003). In addition, CCPs can help the medical team select more cost-effective drugs and shorten the duration of drug treatment, further reducing treatment costs.

### 3.2 Practical review of CCPs work implementation

**FIGURE 2:**
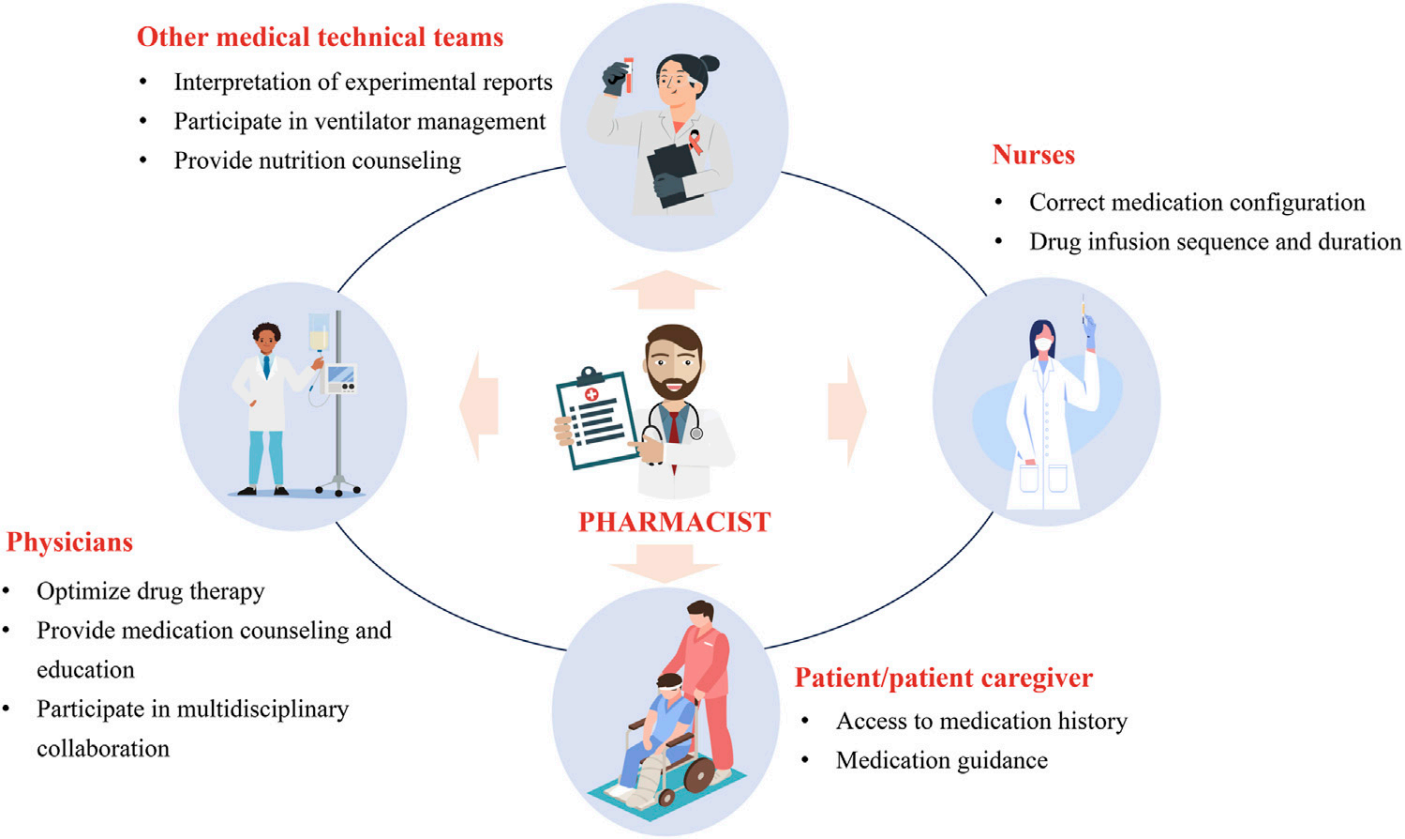
The role of CCPs in supporting multidisciplinary teams.

**FIGURE 3:**
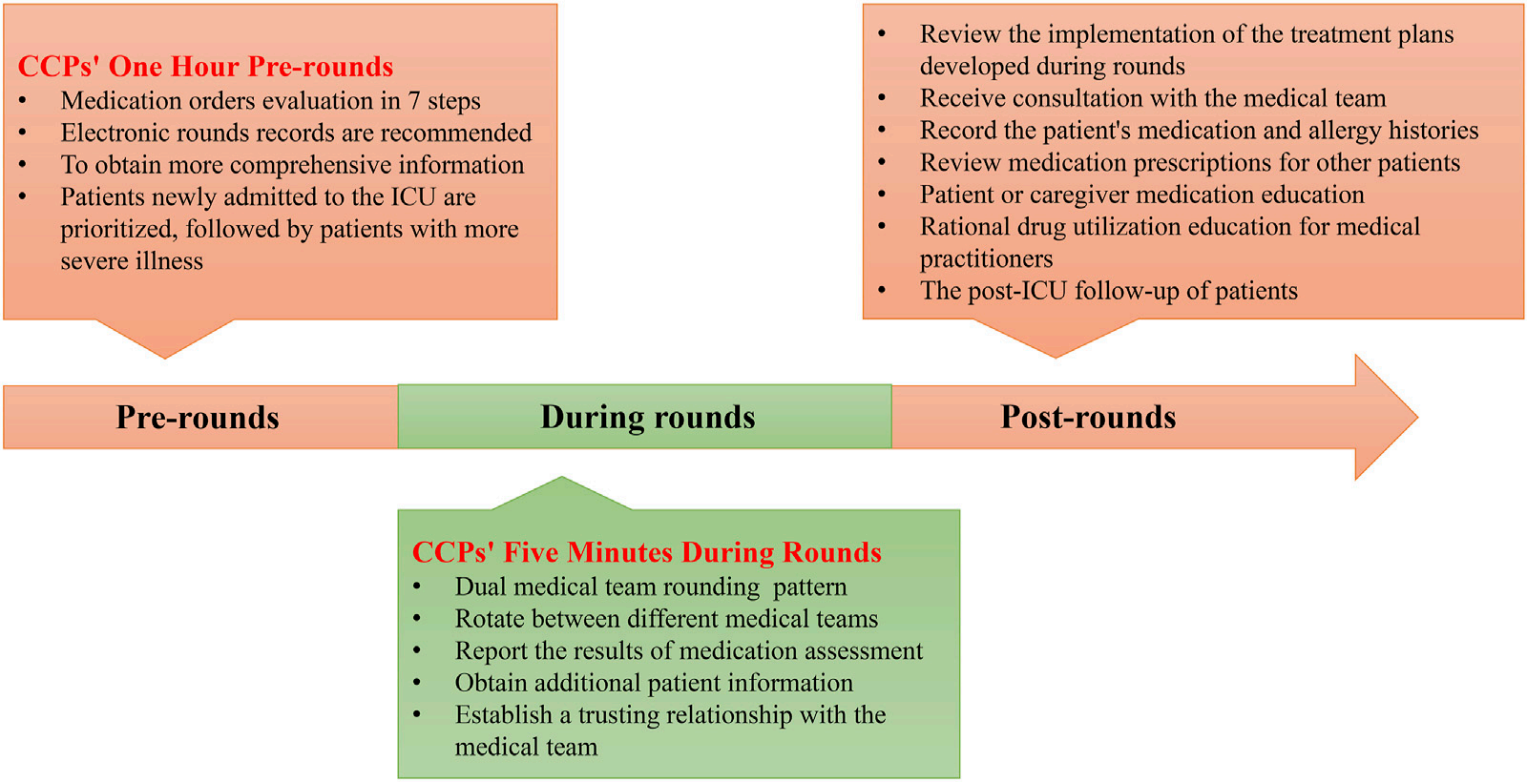
Workflow and key points of critical care pharmacist.

There are various tasks that CCPs can perform, but is there a difference between working as a clinical pharmacist in the ICU and non-ICU settings? Understanding how to effectively implement these various aspects of work in practice is crucial. CCPs can support multiple medical teams, including physicians, nurses, patients/caregivers, and other medical technical teams (Figure 2). Therefore, optimized working patterns can help maximize the value of CCPs. To this end, we have engaged in 10 years of practical exploration and continuous collaboration with the medical team, forming a relatively mature and efficient working pattern to serve patients and improve the quality of medical care. We hope this can provide a reference for more CCPs to enhance their working patterns. We divided the working pattern into three phases: pre-rounds, during rounds, and post-rounds. The details are shown in Figure 3.

#### 3.2.1 CCPs’ one-hour pre-rounds

An essential task before CCPs participate in rounds is the evaluation of medication orders. This process takes at least an hour for a pharmacist to evaluate approximately 10–15 patients and may take longer for less experienced CCPs. Therefore, we call this process the “CCPs’ One Hour Before Rounds.” It is a hectic process, we do not recommend CCPs participate in rounds unprepared, as this may lead to incorrect judgments due to incomplete information. ICU patients may be prescribed up to 20 medications, and accurately recalling the treatment regimen for each patient and the reason behind each specific medication is challenging (Arredondo et al., 2021; Borthwick, 2019; Krzyzaniak and Bajorek, 2017). The condition of ICU patients varies, and an accurate understanding of their condition is essential for pharmacists to develop treatment adjustment plans.

We establish an electronic pharmacist record for each patient, especially those requiring intensive monitoring, allowing for easy highlighting of key points and review. The pre-evaluation of medications enhances the dependability and acceptability of pharmacists’ interventions. The content of MOE includes not only a simple medication review but also requires an individualized drug analysis based on the patient’s specific circumstances to determine a more appropriate medication regimen. This task poses a challenge to the professional expertise of pharmacists. The quality of MOE is often closely related to the depth of CCPs’ involvement in developing treatment plans, which is a crucial aspect of assessing CCPs’ professional competence.

The suggested protocol is to prioritize the evaluation of new patients, followed by those in a more critical condition. To ensure that important questions are not missed, we complete each patient’s MOE using the following seven-step method. Details are shown in Table 1. This seven-step method can be used to perform a comprehensive evaluation of all medical orders in the ICU.

**TABLE 1:** The medication assessment contents of critical care pharmacists.

Category	Content
Step 1: Indication evaluation	• Priority medicines: antibacterial agents, PPIs, albumin, glucocorticoid, high-priced drugs, drugs with poor safety
Step 2: Medication selection and dosage adjustment	• Dosage adjustment: obesity, age, organ dysfunction, extracorporeal life support, shock • Medication selection: such as selection of antimicrobial agents for surgical site infection
Step 3: Course evaluation	• Principle: evidence-based and consideration of individual patient circumstances • Focus on antibacterial agents, PPIs, albumin, glucocorticoid, high-priced drugs, drugs with poor safety
Step 4: Drug-related problems	• MEs: dose error, solvent error, wrong route of administration and incorrect infusion time • DDIs: CYP450 enzyme, P-glycoprotein, monoamine oxidase inhibitors, others (such as valproic acid and carbapenem), the establishment of a dedicated DDI knowledge base is highly recommended • ADEs: abnormal laboratory examination, emerging clinical symptoms (such as fever, epilepsy and arrhythmology)
Step 5: Implementation of TDM	• Select the target drug for TDM • Remind the precautions of TDM • Report interpretation • Adjustment of treatment after TDM
Step 6: Analgesic and sedative dosage	• Overdose warning
Step 7: Pharmaceutical expenses	• Reduce unnecessary medications • Reduce the occurrence of ADEs and MEs • Choose drugs with better cost-effectiveness • Aware of local health insurance reimbursement policies and reduce patient self-payment

##### 3.2.1.1 Whether new therapeutic drugs have indications

The use of multiple medications is often required for ICU patients, and the prevalence of irrational drug administration, particularly antibiotics, is significant (Abdelkarim et al., 2023; Murila et al., 2022). Irrational drug use can lead to an increase in unwanted ADEs and DDIs. The administration of unnecessary medications should be minimized for ICU patients. However, the role of CCPs also extends to reminding physicians to initiate new drug therapies in response to emerging positive outcomes. Therefore, it is imperative for CCPs to thoroughly assess indications for medication use in severe cases, particularly in the case of antibiotics. Medications that lack sufficient indications should prompt doctors to discontinue their usage or minimize treatment duration. We must remember that sometimes “less is more” for seriously ill patients. Rational use of antibiotics is a significant concern (Abdelkarim et al., 2023), and proton pump inhibitors, glucocorticoids, and albumin also deserve attention (Ali et al., 2019; Meng et al., 2017; Zhou et al., 2013). Concurrent use of high-cost medications and narrow therapeutic indices should be approached cautiously.

##### 3.2.1.2 Choose the appropriate medication and dose for critically ill patients

Due to severe inflammation, hypoproteinemia, shock, multiple organ dysfunction, and the need for extracorporeal life support such as continuous renal replacement therapy (CRRT) and extracorporeal membrane oxygenation (ECMO), the processes of drug absorption, distribution, metabolism, and excretion are altered in critically ill patients (Kühn et al., 2020; Roberts et al., 2012; Shaikhouni and Yessayan, 2022). Conventional doses of drugs may not be sufficient for these patients, as they could be either too high or too low (Roberts and Hall, 2013; Veiga and Paiva, 2018). CCPs must have a complete understanding of pathophysiological changes and PK/PD of drugs in critically ill patients to assist physicians in selecting the most appropriate medication and formulating a more rational dose. Choosing the right antibacterial drugs is crucial for patients with special site infections, as incorrect choices may lead to treatment failure.

##### 3.2.1.3 Evaluate the course of medication

Reducing the duration of the drug can lead to a decrease in the number of drug combinations, subsequently minimizing the occurrence of adverse drug reactions. Pharmacists should assist physicians in evaluating the duration of the drug, particularly antibiotics, proton pump inhibitors, and other drugs associated with severe adverse reactions or high costs. The establishment of pharmacist records is crucial in assessing the treatment course. It is important to note that the medication course for critically ill patients may differ from that for non-critically ill patients. Therefore, treatment plans should be evidence-based and tailored to the patient’s actual condition.

##### 3.2.1.4 Detection and analysis of MEs, DDIs, and ADEs

The incidence of MEs and ADRs in ICU patients is exceptionally high (Wilmer et al., 2010). DDIs are often overlooked by physicians, which may increase the incidence of ADRs (Papadopoulos and Smithburger, 2010). CCPs must evaluate MEs, DDIs, and ADRs. The discovery of MEs requires pharmacists to have a comprehensive understanding of all aspects related to drug use, including solvents, routes of administration, and infusion times. Identifying DDIs can be challenging due to the wide variety of medications involved, often leading to their oversight. Examples include interactions related to P-glycoprotein and the cytochrome P450 (CYP450) enzymes, which affect many drugs. However, interactions involving monoamine oxidase inhibitors are often ignored in the ICU. Additionally, the combination of valproic acid and carbapenem presents significant drug interactions that should be noticed.

Pharmacists can establish a dedicated knowledge base for DDIs based on the standard drug list in the local ICU, facilitating the identification of DDIs. Regarding identifying ADEs, pharmacists should assess whether these indicate ADRs when ICU patients present with abnormal changes in laboratory markers, such as elevated creatinine levels or thrombocytopenia, fever of unknown origin, or emerging clinical manifestations, such as seizures. The timely identification and effective management of ADRs are crucial for critically ill patients.

##### 3.2.1.5 Implementation of therapeutic drug monitoring

Even with the participation of pharmacists, empirically prescribed dosing regimens of agents may lead to inadequate or excessive plasma concentrations, resulting in persistently poor clinical outcomes, especially for patients in the ICU. Therapeutic drug monitoring (TDM) can guide dose adjustments to benefit patients (Liu et al., 2023; Loh et al., 2010; Schmid et al., 2023). Pharmacists’ expertise in determining the need for TDM may exceed that of physicians, as they possess specialized knowledge regarding patient profiles and drug characteristics. The CCPs are responsible for developing a TDM plan for the patient and informing the physician of the appropriate timing and precautions for blood draws, such as the volume of blood to be collected. The results of TDM are analyzed and interpreted by pharmacists, who then develop a revised dose adjustment plan.

##### 3.2.1.6 Monitoring the dosage of sedative and analgesic drugs

Excessive use of sedative and analgesic drugs may result in withdrawal syndrome, respiratory depression, fluctuations in heart rate and blood pressure, and can impede the mechanical ventilation weaning process, potentially leading to death (Cho et al., 2020; Gudin et al., 2013; Riker et al., 2009). Pharmacists should have a comprehensive understanding of the PK/PD characteristics associated with analgesic and sedative drugs, allowing them to formulate more appropriate treatment plans for patients and effectively monitor the administration of these medications to prevent potential overdose. The dosage of analgesic and sedative drugs should be limited according to established guidelines (Devlin et al., 2018; Smith et al., 2022).

##### 3.2.1.7 Rein in pharmaceutical expenses

The participation of pharmacists can significantly enhance the cost-benefit relationship for ICU patients (MacLaren et al., 2008). The improvement of economic impact manifests itself primarily in three aspects. First, the involvement of pharmacists reduces unnecessary drug use. Second, pharmacists reduce treatment costs by reducing the incidence of ADEs and MEs. Lastly, pharmacists assist physicians in selecting more cost-effective medications. Therefore, CCPs must completely understand pharmacoeconomics to choose more cost-effective options. Additionally, they should be familiar with local health insurance reimbursement policies to minimize patient self-payment.

#### 3.2.2 Responsibilities of the pharmacist during rounds

Patient rounds are significant, and relying solely on electronic medical records for information is insufficient. Additional insights are collected through active participation in rounds. To ensure physicians fully understand the outcomes of the pharmacist’s MOE, we present the MOE results within the first 5 minutes of rounds, a segment we refer to as the “CCPs’ Five Minutes During Rounds.” These 5 minutes summarize the pharmacist’s professional work and should articulate the rationale for any proposed interventions. During rounds, medical teams discuss the CCPs’ recommendations in the context of each patient’s situation and decide whether to adopt these suggestions. Additionally, pharmacists should gather more information from physicians and nurses to improve the content of medication evaluations. Rounding facilitates effective communication between pharmacists and medical teams and helps establish mutual trust. Although rounds in the ICU can be time-consuming and indispensable, even in developed countries, the number of CCPs is limited, making it impossible to provide coverage for all critically ill patients. Our CCPs rotate between different medical teams and consider implementing a “dual medical group rounding pattern.” For example, pharmacists conduct rounds with “medical group A″ on Mondays, Wednesdays, and Fridays and with “medical group B” on Tuesdays and Thursdays. This approach optimizes the provision of pharmaceutical care.

#### 3.2.3 Regular work of CCPs post-rounds

Participation in rounds may account for approximately 40% of a pharmacist’s working hours, which includes the time spent on MOE and the rounds themselves. However, pharmacists can perform numerous specialized tasks after rounds, which we list below.

##### 3.2.3.1 Review the implementation of the treatment plans developed during rounds

ICU patients often face several changes to their daily treatment plans, including both drug and non-drug treatment strategies. CCPs are responsible for ensuring that adjustments related to drug treatment are implemented after rounds. Therefore, after the rounds, a brief review of the patient’s medical orders is required to confirm that all modifications have been accurately performed.

##### 3.2.3.2 Receive a consultation with the medical team

CCPs are available during working hours and are often accessible outside of these times because emergencies may require the temporary use of special drugs. Pharmacists must be ready to respond to inquiries from doctors and care teams regarding aspects such as drug dosages and administration methods.

##### 3.2.3.3 Record the patient’s medication and allergy histories

CCPs play a vital role in helping doctors communicate effectively with patients or their caregivers, understand patient medical histories, including previous medications and allergy records, and assess potential allergy risks. For example, doctors must know that individuals allergic to sulfanilamide may also react to furosemide, a fact that is often overlooked.

##### 3.2.3.4 ICU full patient medication review

Despite limited workforce resources, we aim to establish “all-patient pharmaceutical care” for ICU patients. Even with the implementation of the “dual medical team rounding pattern,” pharmaceutical care is still insufficient to meet the needs of all patients. Therefore, the scope of pharmacist drug assessments should be extended to include all patients who are not part of the round groups. At least twice a week, CCPs should conduct comprehensive prescription medication assessments for patients who do not participate in rounds. Although many medical institutions have adopted electronic prescription review systems to intercept MEs, numerous MEs cannot be effectively prevented by these systems alone (Osmani et al., 2023; Roumeliotis et al., 2019; Vejdani et al., 2022). Consequently, manual review by pharmacists remains indispensable.

##### 3.2.3.5 Patient or caregiver medication education

Medication education for critically ill patients is often not implemented due to the administration of deep sedation. However, when ICU patients are transferred out of the ICU, they frequently require multiple medications to maintain their treatment. Pharmacists can educate patients or their caregivers on essential medicines to improve treatment adherence during this transition period. However, it is challenging for pharmacists to inform all patients transferred from the ICU. Therefore, implementing targeted medication education systems can effectively address this issue. For example, medication education could be prioritized for patients who need more than five medications upon transfer from the ICU or for those whose organ functions have not fully recovered.

##### 3.2.3.6 Rational drug utilization education for medical practitioners

CCPs can provide a comprehensive overview of prevalent MEs and DDIs to facilitate training healthcare professionals, including physicians and nurses. This role is particularly vital in teaching hospitals, where frequent rotation of medical and nursing students may contribute to an increased incidence of MEs.

##### 3.2.3.7 Post-ICU follow-up of patients

An increasing number of people are discharged from the ICUs for ongoing treatment (Svenningsen et al., 2017). Studies have shown that post-ICU follow-up is efficacious in improving patient mental health and alleviating depressive symptoms (Rosa et al., 2019). We propose that patients discharged from the ICU, particularly those requiring multi-drug therapy, undergo pharmaceutical follow-up. This strategy can effectively evaluate treatment continuity and adherence while facilitating medication simplification as needed. However, following up with all patients after ICU is not feasible. Therefore, we suggest establishing criteria to prioritize patients for follow-up. Each pharmacist can customize the follow-up protocol based on their working capacity and available time. Our primary focus for ICU follow-up is on patients with impaired organ function and those on multi-drug regimens to reduce their risk of returning to the ICU due to drug-induced injuries.

## 4 Special program of CCPs

### 4.1 Multidisciplinary management of antimicrobial drugs

Overusing antimicrobial agents is expected in the ICU as physicians strive to achieve early and appropriate empiric antimicrobial therapy to improve patient outcomes (Murphy et al., 2022). It is noteworthy that up to half of ICU patients receiving empirical antibiotic therapy have no definitively confirmed infection, and strategies such as de-escalation and shortened treatment durations are insufficiently considered for those with documented sepsis (Timsit et al., 2019). In our hospital, CCPs lead a multidisciplinary discussion group focused on the rational use of antimicrobials. This group meets weekly (every Thursday) to discuss cases involving patients using more than three antimicrobials. Participants include senior ICU physicians, CCPs, and microbiologists. This collaborative approach helps physicians optimize antimicrobial therapy with multidisciplinary support and encourages them to reconsider the need for ongoing antimicrobial treatments and discontinue unnecessary antibiotics. We plan to report in more detail on the patterns and successes of this initiative in the future.

### 4.2 Participate in the outpatient post-ICU service

An increasing number of patients treated in the ICU survive to discharge. Outpatient follow-up for these patients is crucial to providing continuity of care and screening for post-ICU complications (Wilbur et al., 2021). Pharmacists play a vital role in this outpatient follow-up by assessing whether patients leave the ICU with unrecovered organ dysfunction, whether medication doses need adjustment, and whether the duration of the medication is adequate. Pharmacists educate patients about lifestyle choices and proper drug use to improve adherence and reduce drug-induced injuries.

## 5 Discussion

This article represents the first comprehensive review of the function of CCPs. It intends to highlight the significant impact CCPs can have in various patient care areas. The aim is to guide CCPs in establishing an operational framework that optimizes their participation in patient care processes. A pharmacist has multiple critical tasks to complete before, during, and after rounds. The process of “CCPs’ One Hour Before Rounds” is vital for a comprehensive evaluation of medication utilization, utilizing the seven-step method discussed, which effectively prevents potential inappropriate medication therapy. The “CCPs’ Five Minutes During Rounds” ensure the pharmacist’s medication recommendations are fully understood and implemented. Participation in rounds also gathers additional information from physicians and nurses and establishes trust within the medical team.

Given the often-limited number of CCPs, we recommend extending pharmaceutical care to as many patients as possible. Implementing a “dual medical team rounding system” in combination with an “ICU full patient medication review” could establish comprehensive pharmaceutical care for all ICU patients. Beyond the rounds, the pharmacist’s role is crucial in conducting medication reviews that decrease the occurrence of DDIs and MEs. The special management of antimicrobial drugs can also promote the rational use of antibiotics.

Post-ICU follow-up, medication education for patients or caregivers, and education for medical practitioners are vital roles that CCPs can assume. The described work patterns may be applicable to non-CCPs; however, it is crucial that CCPs develop a rationalized and standardized workflow, especially given the complexities of ICU patient care.

The requirements for CCPs vary across different countries due to disparities in development and policies. Firstly, research indicates that the presence of clinical pharmacists in approximately 92.6% of ICUs in the United States surpasses that in low-to-middle income countries (Gershengorn et al., 2024). In the United Kingdom, a certain number of ICUs are staffed with pharmacists possessing independent prescribing authority (Reid et al., 2018). The action is prohibited by Chinese legislation. Concurrently, Chinese CCPs are also entrusted with a myriad of responsibilities encompassing policy and institution, including the prescription review after the fact. For instance, the reasonableness of drug utilization during hospitalization was assessed after the patient’s discharge. However, the primary responsibility of CCPs in any country lies in ensuring the safety and efficacy of drug use for patients, promoting rational drug utilization, and controlling the cost of pharmacotherapy.

The value of CCPs deserves recognition, and the role places stringent professional demands on practitioners. The growth of CCPs often requires a prolonged period to accumulate substantial experience and knowledge. Implementing this operational mode is expected to accelerate the development of CCP skills. We propose that pharmacists tailor their work to the specific needs of admitted patients and advocate for increased policy support to promote CCP advancement.
